# Supplementary material for: Causal Relationship between Aging and Anorexia Nervosa: A White-Matter-Microstructure-Mediated Mendelian Randomization Analysis
Source: Biomedicines. 2024 Aug 16;12(8):1874. doi: 10.3390/biomedicines12081874 (PMC11351342; doi:10.3390/biomedicines12081874)
Supplement: Supplementary file 1 [file biomedicines-12-01874-s001.zip › Supplementary Figure/Figure S8/Leaveoneout plot of MR analysis for TL to ubm-b-2059.pdf]

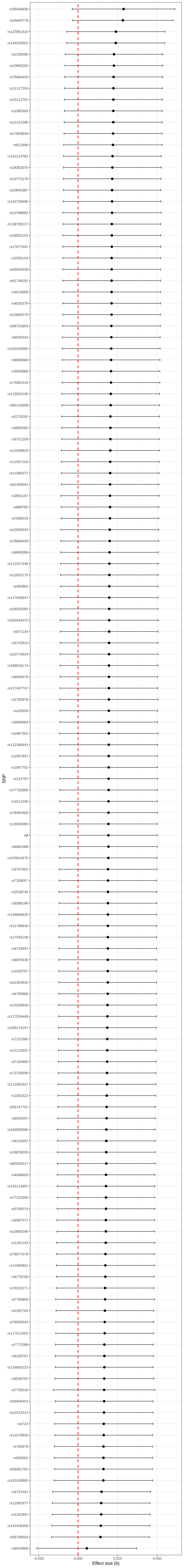

rs35446936
rs35640778
rs137901416
rs144204502
rs2230590
rs10905255
rs75664430
rs11117354
rs10112752
rs1985369
rs11412296
rs17803849
rs611646
rs141214782
rs28363070
rs10773176
rs10845387
rs142730696
rs10768683
rs139795227
rs28502153
rs17677991
rs2555104
rs45604339
rs61748181
rs4616688
rs4530278
rs10840270
rs66731853
rs6590343
rs150150565
rs6659669
rs3093888
rs73581419
rs113525195
rs80116508
rs2276182
rs9955360
rs6751209
rs11699829
rs11557154
rs11085072
rs61405042
rs3891167
rs869785
rs7099229
rs12925933
rs76666449
rs9940099
rs111527438
rs12932179
rs450962
rs117630647
rs34550383
rs202034370
rs871134
rs5742915
rs10774624
rs188918174
rs6584579
rs117407747
rs2763979
rs429358
rs6669563
rs2967355
rs112394943
rs1957937
rs1907702
rs131797
rs77732866
rs1611236
rs78491606
rs13062095
All
rs6881568
rs376641875
rs3767952
rs7209057
rs2538745
rs9398196
rs139669835
rs11769630
rs17445108
rs4743037
rs9878436
rs1023767
rs41304832
rs6790988
rs13230646
rs117034449
rs185174247
rs7221585
rs11212631
rs7164950
rs73730598
rs111950327
rs1003322
rs55747751
rs6054257
rs182059586
rs6102497
rs16978028
rs80324517
rs4498805
rs145114957
rs77231040
rs3785074
rs6587577
rs10805346
rs1291143
rs79977579
rs11584821
rs6776756
rs76219171
rs7790856
rs2282764
rs76065543
rs117512405
rs7772289
rs8105767
rs116863223
rs6536702
rs7705526
rs59409453
rs12412214
rs4724
rs11579626
rs762679
rs932002
rs56061761
rs143190905
rs4731541
rs11991877
rs1332941
rs142426306
rs56799654
rs9419958
SNP
-0.025
0.000
0.025
0.050
Effect size (b)
